# Exogenous C_8_-Ceramide Induces Apoptosis by Overproduction of ROS and the Switch of Superoxide Dismutases SOD1 to SOD2 in Human Lung Cancer Cells

**DOI:** 10.3390/ijms19103010

**Published:** 2018-10-02

**Authors:** Yuli C. Chang, Yao Fong, Eing-Mei Tsai, Ya-Gin Chang, Han Lin Chou, Chang-Yi Wu, Yen-Ni Teng, Ta-Chih Liu, Shyng-Shiou Yuan, Chien-Chih Chiu

**Affiliations:** 1 Department of Laboratory Medicine, Kaohsiung Medical University Hospital, Kaohsiung Medical University, Kaohsiung 807, Taiwan; yuli33@ms28.hinet.net (Y.C.C.); d730093@cc.kmu.edu.tw (T.-C.L.); 2 Chest Surgery, Chi-Mei Medical Center, Yung Kang City, Tainan 901, Taiwan; yaou.fong@msa.hinet.net; 3 Graduate Institute of Medicine, College of Medicine, Kaohsiung Medical University, Kaohsiung 807, Taiwan; tsaieing@yahoo.com; 4 Department of Obstetrics and Gynecology, Kaohsiung Medical University Hospital, Kaohsiung 807, Taiwan; 5 Department of Biotechnology, Kaohsiung Medical University, Kaohsiung 807, Taiwan; yaginchang@hotmail.com.tw (Y.-G.C.); d992050005@student.nsysu.edu.tw (H.-L.C.); cywu@mail.nsysu.edu.tw (C.-Y.W.); 6 Department of Biological Sciences, National Sun Yat-Sen University, Kaohsiung 804, Taiwan; 7 Department of Biological Sciences and Technology, National University of Tainan, Tainan 700, Taiwan; tengyenni@mail.nutn.edu.tw; 8 Institute of Clinical Medicine, College of Medicine, Kaohsiung Medical University Hospital, Kaohsiung 807, Taiwan; 9 Translational Research Center, Cancer Center, Department of Medical Research, Department of Obstetrics and Gynecology, Kaohsiung Medical University Hospital, Kaohsiung Medical University, Kaohsiung 807, Taiwan; yuanssf@ms33.hinet.net; 10 Research Center for Environment Medicine, Kaohsiung Medical University, Kaohsiung 807, Taiwan

**Keywords:** lung cancer, apoptosis, C_8_-ceramide, ROS, SOD switch, cyclin D1

## Abstract

Ceramides, abundant sphingolipids on the cell membrane, can act as signaling molecules to regulate cellular functions including cell viability. Exogenous ceramide has been shown to exert potent anti-proliferative effects against cancer cells, but little is known about how it affects reactive oxygen species (ROS) in lung cancer cells. In this study, we investigated the effect of *N*-octanoyl-*D*-erythro-sphingosine (C_8_-ceramide) on human non-small-cell lung cancer H1299 cells. Flow cytometry-based assays indicated that C_8_-ceramide increased the level of endogenous ROS in H1299 cells. Interestingly, the ratio of superoxide dismutases (SODs) SOD1 and SOD2 seem to be regulated by C_8_-ceramide treatment. Furthermore, the accumulation of cell cycle G1 phase and apoptotic populations in C_8_-ceramide-treated H1299 cells was observed. The results of the Western blot showed that C_8_-ceramide causes a dramatically increased protein level of cyclin D1, a critical regulator of cell cycle G1/S transition. These results suggest that C_8_-ceramide acts as a potent chemotherapeutic agent and may increase the endogenous ROS level by regulating the switch of SOD1 and SOD2, causing the anti-proliferation, and consequently triggering the apoptosis of NSCLC H1299 cells. Accordingly, our works may give a promising strategy for lung cancer treatment in the future.

## 1. Introduction

Lung cancer is a leading cause of cancer death worldwide, including in the Taiwan region. Small cell lung cancer (SCLC) and non-small-cell lung cancer (NSCLC) are the primary types of lung cancer. About 90% of lung cancer cases are diagnosed at an advanced stage where treatment is not available [1,2,3,4]. Approximately 80% of lung cancer belongs to NSCLC histologically [5]. The progression of the epithelial cells with undetermined clinical significance affects cell proliferation, apoptosis, angiogenesis, and activation of oncogenes [6,7]. Because NSCLC easily metastasizes into nearby tissues or other parts of the body, NSCLC patients have a relatively high mortality rate, and therefore, an effective treatment for NSCLC patients is urgent [8].

Ceramides, composed of fatty acid and sphingosine, are abundant sphingolipids on the cell membrane. Ceramides have been reported to act as signaling molecules to regulate cellular functions [9], including cellular proliferation and viability [10]. Additionally, it was well-documented that ceramides act as essential mediators in apoptosis pathways [11,12]. Exogenous ceramides have been reported as anti-cancer potential chemotherapeutics in malignancies, including pancreatic [13], breast [14], gastric [15], and hematologic [16] cancer cells. Furthermore, a previous study showed that exogenous ceramides sensitized gemcitabine-induced premature senescence [17]. Studies regarding exogenous short carbon-chain ceramides such as C_2_- or C_6_-ceramides were well-documented [12,14,18,19]. In contrast, the bioactivities of long carbon-chain ceramide were less addressed except some skin disease being used [20], because of its limited membrane permeability [21].

Superoxide dismutases (SODs), endogenous reactive oxygen species (ROS) scavenging enzymes, involves the regulation of ROS level in cells. There are three types of SODs, SOD1 (CuZn-SOD) is found in intracellular cytoplasmic spaces, SOD2 (Mn-SOD) is primarily found in the mitochondrial spaces and SOD3 (EC-SOD) is found in extracellular spaces [22]. SOD1 overexpression is frequently observed in many cancers [23], whereas SOD2 is downregulated [24]. SOD1 inhibition is considered to induce cell death potentially [25], and targeting SOD1 in lung cancer has been reported [11]. Furthermore, the expression ratio of SOD1/SOD2 seems to be a switch and plays an essential physiological role in breast cancer cells [26]. Therefore, the modulation in the ratio of SOD1/SOD2 may be a promising strategy for treating cancer cells.

Cyclin D1, an abundant protein in the G1 phase of the cell cycle, can induce global transcriptional downregulation in lymphoid neoplasms [27], and also can be critical for proliferating cells in G1/S transition [28,29]. Deregulation of cyclin D1 has been reported to be observed in cancers including breast cancer and lung cancer cells [30,31]. Cyclin D1 has been thought to promote the cellular proliferation and survival. Cyclin D1 composes a feedback loop of positively contributing to tumor growth in gastric cancer reported by Hayakawa et al. [32], and cyclin D1 links the signal of extracellular environment to upregulate proliferation of cancers including prostate cancer [33], breast cancer [34], and bladder cancer [35]. The proliferation [36] and invasion [37] of lung cancer cells can be promoted by regulating the expression of cyclin D1. However, the overexpression of cyclin D1 was also reported to be correlated with apoptosis under specific concomitant signals of arrest such as serum starvation or proliferation arrest [38]. For example, the increase of cyclin D1 artificially is sufficient to induce the apoptosis both in neural and non-neural cell types [39]. Additionally, it was also reported that dysfunction of cyclin D1 induces apoptosis of rat pheochromocytoma PC12 cells [40], and the expression level changes of cyclin D1 are related to apoptosis via G1 arrest of the cell cycle in gastric cancer cells [32].

In the study, we examined the growth inhibitory effects of C_8_-ceramide in NSCLC H1299 cells. Besides, the underlying mechanism of apoptosis, especially the role of cyclin D1 and the regulation of SOD1 and SOD2 was also discussed.

## 2. Results

### 2.1. C_8_-ceramide Exerts the Anti-Proliferation Potential against H1299 Lung Cancer Cells.

**Figure 1:**
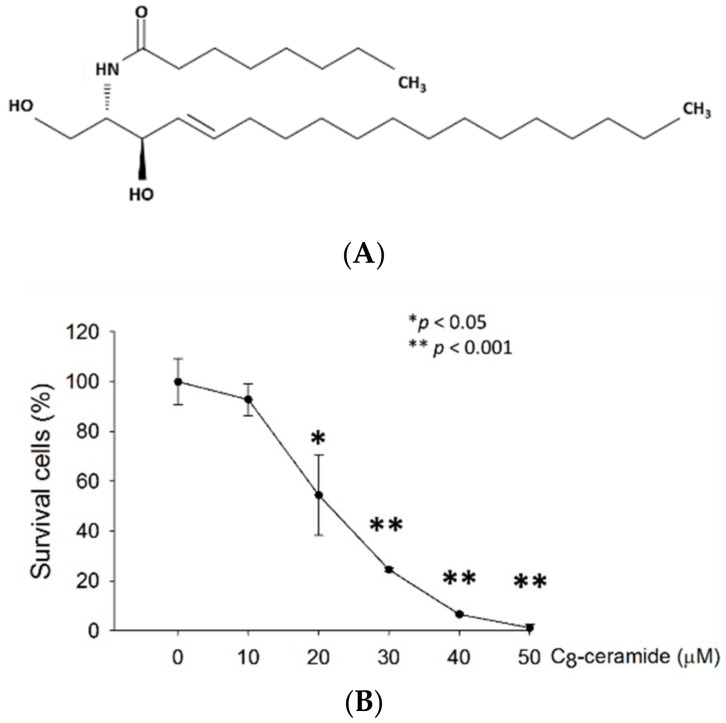
(**A**) Structure of the C_8_-ceramide (*N*-octanoyl-d-erythro-sphingosine). (**B**) The quantitative analysis of cell proliferation assay showed the inhibition of growth in a dose-dependent manner (* *p* < 0.05, ** *p* < 0.001 for C_8_-ceramide treatment versus respective control).

H1299 lung cancer cells were treated with increasing concentrations of C_8_-ceramide for 24 h, and proliferation rates were determined using Trypan blue assay. The rate of cellular proliferation of C_8_-ceramide-treated H1299 lung cancer cells decreased in a dose-dependent manner. The 50% inhibitory concentration (IC_50,_ 24 h) of C_8_-ceramide for H1299 cells was 22.9 µM (Figure 1B).

### 2.2. C_8_-Ceramide May Cause the G1 Arrest of H1299 Cells

**Figure 2:**
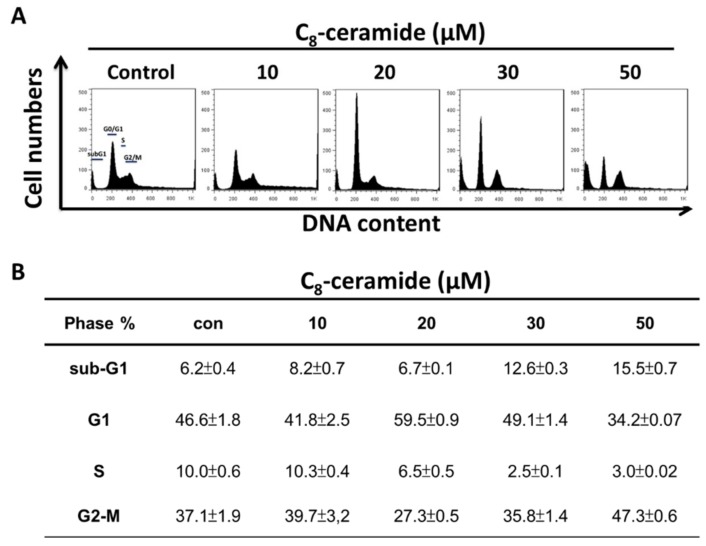
C_8_-ceramide-induced cell arrest of G1 in H1299 cells. Cells were treated with indicated concentrations (from 10 to 50 µM) of C_8_-ceramide for 24 h respectively. (**A**) Representative cell cycle distribution in C_8_-ceramide-treated H1299 cells. **(B)** The results of quantitative analysis. C_8_-ceramide induces the apoptosis of H1299 cells in a dose-dependent manner.

The cell cycle profiles of C_8_-ceramide treated H1299 lung cancer cells was examined using flow cytometry. Cells accumulated in the G1 phase when treated with 20 and 30 µM C_8_-ceramide. An increased sub-G1 population was observed at 30 and 50 µM C_8_-ceramide treated cells (Figure 2).

**Figure 3:**
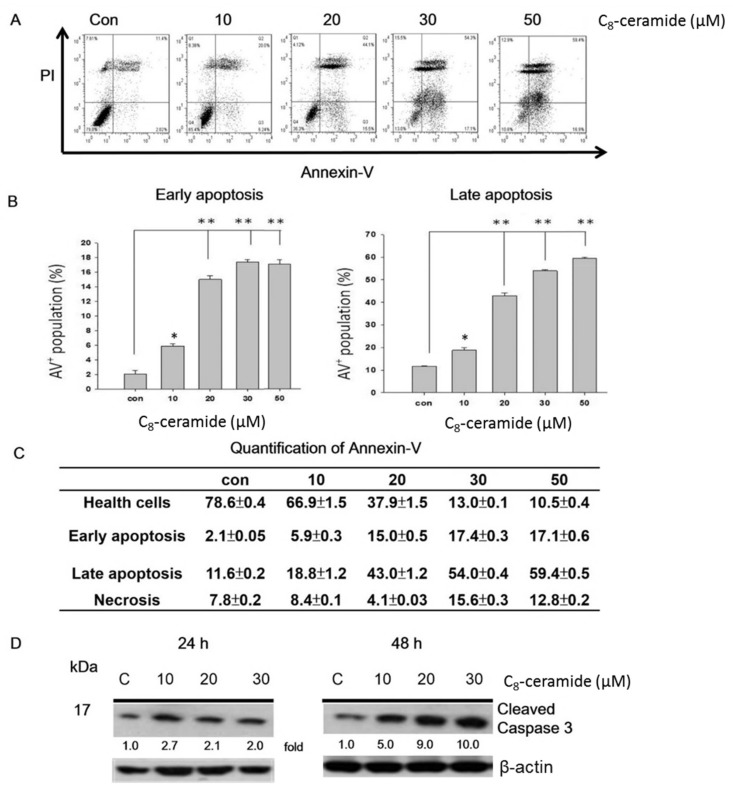
C_8_-ceramide-induced apoptotic profiles of lung cancer H1299 cells. Cells were treated with indicated concentrations (from 10 to 50 µM) C_8_-ceramide for 24 h and 48 h respectively. (**A**) Representative profiles of apoptosis detected by Annexin V/PI double staining in C_8_-ceramide-treated H1299 cells for 48 h. (**B**) Population assessment of early and late-stage apoptosis. * *p* < 0.05, ** *p* < 0.001 for C_8_-ceramide treatment versus respective control. (**C**) The results of the quantitative analysis for apoptosis population (%). Data, mean ± SD (*n* = 3). (**D**) The proteolytic activation (cleaved form) of caspase-3 in C_8_-ceramide treated H1299 cells. β-actin as an internal control.

In Figure 3A, the profiles of Annexin V/PI -positive percentages were shown for the treatments with vehicle control (0.5% DMSO) or indicated concentrations (from 10 to 50 µM) of C_8_-ceramide for 48 h respectively. After 48 h of the C_8_-ceramide treatment, the Annexin V-positive percentages of H1299 cells rose in a dose-dependent manner, and the level of cleaved caspase-3 was shown (Figure 3B,C).

### 2.3. The Detection of Endogenous ROS in C_8_-Ceramide-Treated H1299 Cells

**Figure 4:**
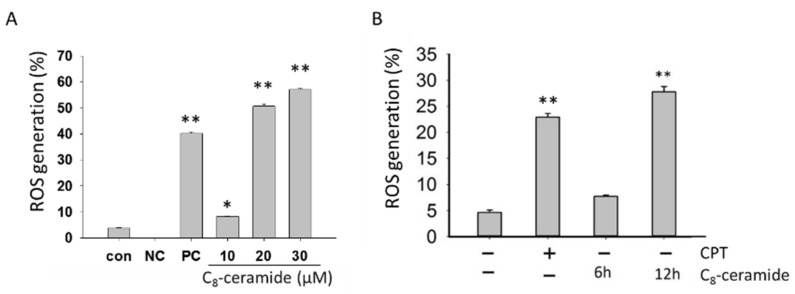
C_8_-ceramide increases the level of ROS in H1299 cells. (**A**) Flow cytometry-based ROS assessment for C_8_-ceramide-treated cells. Cells were treated with indicated concentrations (from 0 to 30 µM) of C_8_-ceramide for 24 h respectively. Positive % was indicated in each panel. PC: positive control, 1 mM H_2_O_2_. CON: vehicle control. NC: negative control, unstained cells. Quantitative analysis. Data presented as mean ± S.D. in triplicate. Asterisks indicated statistically significant differences compared with those of the control (* *p* < 0.05 and ** *p* < 0.001 for control versus C_8_-ceramide treatment respectively). (**B**) The quantitative analysis. Data presented as mean ± S.D. in triplicates. Five μM of camptothecin (CPT) as a positive control. Asterisks indicated statistically significant differences compared with those of the control (** *p* < 0.001 for C_8_-ceramide treatment versus respective control in 6 and 12 h).

To explore whether C_8_-ceramide affects the endogenous ROS level of H1299 cells, we analyzed ROS generation of C_8_-ceramide-treated H1299 cells using flow cytometer-based 2′,7′-dichlorofluorescein diacetate (DCFDA) staining. The changes in endogenous ROS level by C_8_-ceramide treatment for 24 h were shown (Figure 4A). The levels of endogenous ROS were significantly increased in H1299 cells in a dose-dependent manner (* *p* < 0.05 and ** *p* < 0.001) following C_8_-ceramide treatment (** *p* < 0.001) (Figure 4B).

### 2.4. Assessment of Migration in C8-ceramide-treated H1299 cells

**Figure 5:**
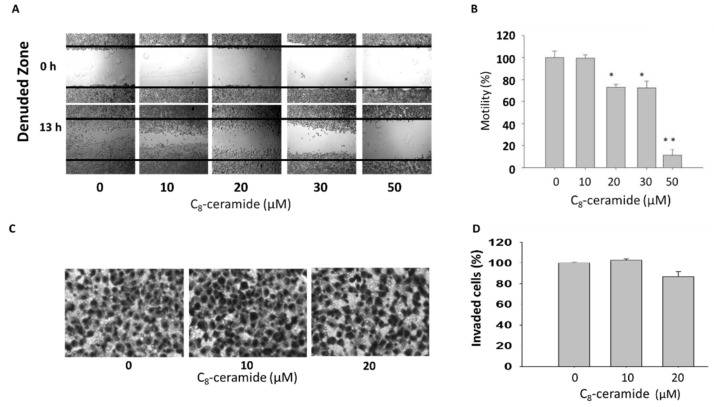
The effects of C8-ceramide on the migration and invasion of H1299 lung cancer cells. (**A**) A confluent culture of H1299 cells was seeded onto a 12-well plate, and cells have created a gap with a 200 µL tip. The cells were treated with indicated concentrations (from 0 to 50 µM) of C_8_-ceramide for 24 h respectively. (**B**) Quantitative analysis of (**A**) (* *p* < 0.05 and ** *p* < 0.001 for C_8_-ceramide treatment versus respective control). (**C**) Boyden’s transwell assay was conducted to examine the effect of C_8_-ceramide on the invasion of H1299 cells. (**D**) Quantitative analysis of (**C**) Magnification: 100×.

To examine whether C_8_-ceramide affects the cellular migration, a critical index of cancer metastasis, the wound healing assay was conducted. Image panel shows the results of wound healing assay and Boyden’s transwell assay (Figure 5). As shown in Figure 5A,B, the results showed the moderately inhibitory effect of C_8_-ceramide on the migration of H1299 cells, whereas the no significant changes were observed when we further assessed the anti-migration effect of C_8_-ceramide, showing that sub-IC_50_ dose (below 20 μM) of C_8_-ceramide is ineffective to suppress the invasion of H1299 lung cancer cells (Figure 5C,D). Therefore, the results suggesting that C_8_-ceramide induces anti-proliferation and apoptosis rather than anti-migration and anti-invasion in NSCLC cancer cells.

### 2.5. The Modulation of SOD1 and SOD2 in C_8_-Ceramide Treated H1299 Cells

**Figure 6:**
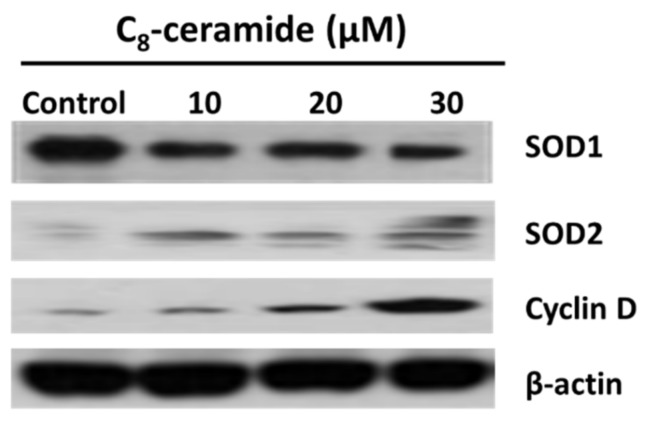
Regulation of SOD1/2 and cyclin D1 proteins induced by C_8_-ceramide. After C_8_-ceramide treatment, SOD1 downregulation may be controlled by ROS, which can negative feedback to SOD1 switched SOD2 and then modulated the expression of cyclin D1. β-actin as an internal control.

The C_8_-ceramide-induced treatment modulated the levels of SOD1 and cyclin D1 in H1299 lung cancer cells on a protein level, which was examined by Western blotting in the present study. Both SOD1 (decreased) and cyclin D1 (increased) levels in C_8_-ceramide-treated H1299 cells were significantly changed at the concentration of 20 and 30 µM (Figure 6). In contrast, the protein levels of SOD2 were upregulated dramatically (Figure 6).

## 3. Discussion

The modulations of ceramides as the strategy for lung cancer therapies have been reported [12,14,41]. Both exogenous and endogenous ceramides have been reported to play essential roles in the apoptotic death of cancer cells induced by ionizing radiation [42] or chemotherapeutic agents [43,44]. However, little is known regarding the effects of long carbon-chain ceramides [45]. Among the subtype of NSCLC cells, large cell carcinoma has been reported to exhibit higher invasiveness, and it is difficult for treatment [46,47]. Therefore, we first used H1299 cells, a cell line of large cell carcinoma to examine the anti-lung cancer activity of C_8_-ceramide in the study. We also observed the correlations of ROS and SOD expression in lung cancer H1299 cells following exogenous C_8_-ceramide treatment.

The modulation of endogenous ROS are essential for cellular survival and proliferation [48] and increased ROS level could provide cancer cells with advantages of survival and growth [49]. However, excessive oxidative stresses may cause cell death. A previous study showed that superoxide dismutase SOD1 acts as an endogenous ROS scavenger and a potential contributor to the survival of the cancer cell under conditions of high oxidative stress [50]. In the study, our results showed that the C_8_-ceramide treatment causes the decreased expression of SOD1 concomitantly increased the level of ROS stress.

Cyclin D1 has been thought to be oncogenic [51], and cyclin D1 overexpression was frequently observed in cancers. In addition, the upregulated expression of cyclin D1 promotes the proliferation of HT29 human colon cancer cells [52]. Crebanine, an aporphine alkaloid, was reported to exert an anti-cancer activity through down-regulating cyclin D1 expression in lung adenocarcinoma A549 [53]. On the contrary, cyclin D1 overexpression was also reported to be correlated with apoptosis under specific concomitant signals of arrest such as serum starvation or proliferation arrest [38], which may due to the feedback loop between cyclin D1 and tumor suppressors. Recently, Sun’s work suggested that cyclin D1 is required for miRNA let-7-induced cancer repression and the cell death [54].

Papa and Manfredi showed that cancer cells have elevated levels of reactive oxygen species (ROS), which are generated by modulating superoxide dismutase (SOD) as an essential antioxidant enzyme [25]. Furthermore, the activity of SOD2 was decreased to 87% of breast cancer cases, whereas the levels of SOD1 and ROS were upregulated. The ratio of SOD2 to SOD1 seems to be critical for maintaining endogenous ROS in cells. Therefore, the switch mechanism of SOD1 to SOD2 may play a significant role in cellular physiology, such as invasion or proliferation of breast cancer cells. Consistently, the decrease of SOD1 and SOD2 ratio was also observed in H1299 cells following C_8_-ceramide treatment, suggesting that the anti-lung cancer effects of C_8_-ceramide may be closely correlated with the mechanism of SOD1 to SOD2 switch.

Recent studies regarding cell apoptosis showed the biological correlation between cyclin D1 and SOD1 in various human diseases, including genetic diseases and cancers [28,31,55]. The cell proliferation and cell cycle are regulated by directly interacting with cyclin D1. Cyclin D1 was up-regulated when it was involved in neurodegenerative processes related to SOD1 [30]. Furthermore, recent evidence strongly suggested the relationship between SOD and cyclin D, for example, a cell cycle signaling that is cyclin D at the neuronal G1-S checkpoint may be critical for the neuronal cell death caused by mutant SOD1 [28].

However, according to the results of those above studies, likewise, our findings suggest that C_8_-ceramide causes a high level of ROS that was contributed by down-regulated SOD1 and up-regulated cyclin D to promote cell cycle G1 arrest, the growth inhibition, and apoptosis of H1299 cells. Our results indicate that a cell cycle signaling changed by the up-regulation of ROS may reconstitute a critical step in the cell death pathway caused by SOD1 and cyclin D1, which was treated by exogenous long carbon-chain C_8_-ceramide.

Importantly, the results of our study showed that C_8_-ceramide induces apoptosis of H1299 cells, indicating the potential of C_8_-ceramide against human lung cancer cells. Menon et al.’s work investigated the regulatory role of SOD2 (MnSOD) and cyclin D1 in *N*-acetyl-*L*-cysteine (NAC)-induced G1 phase arrest in mouse fibroblast [56], and their results suggested that NAC upregulates endogenous O_2_ pathway and induces G1-arrest through both increasing SOD2 activity and decreasing cyclin D1 [56]. However, our results showed both SOD2 and cyclin D1 are significantly accumulated in C_8_-ceramide-induced G1-arrest, suggesting the cell types and drug treatments cause the different mechanism from Menon’s work.

**Figure 7:**
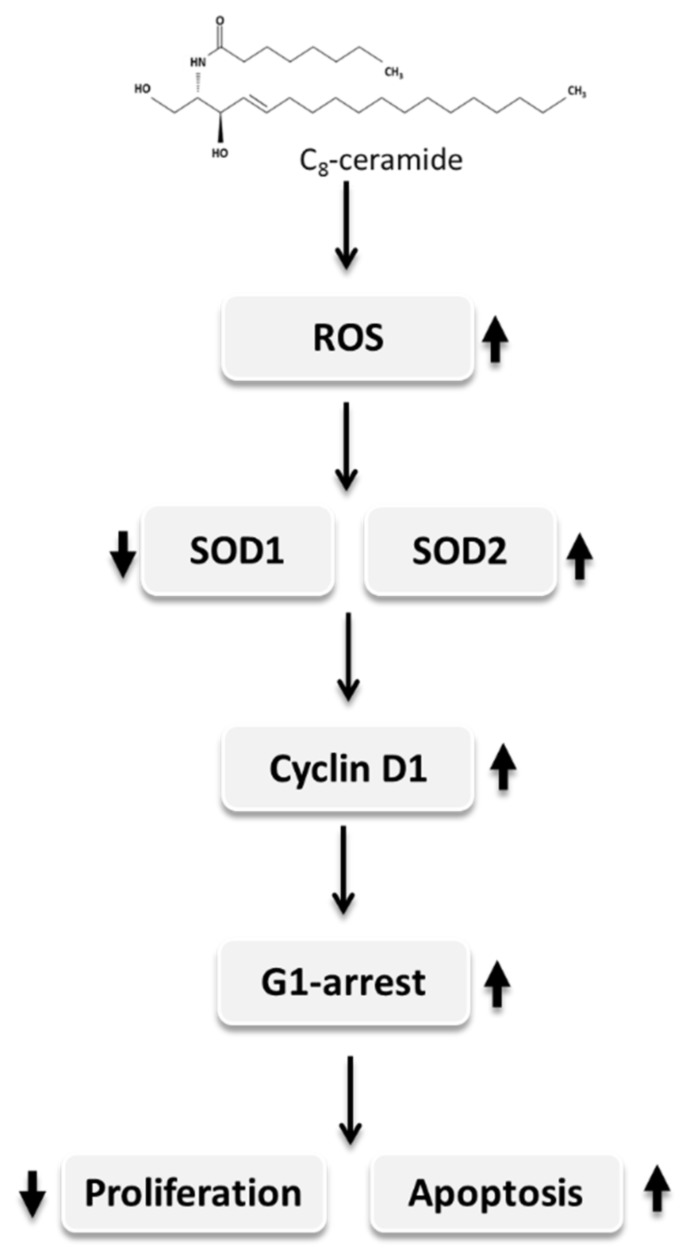
A proposed model of C_8_-ceramide-induced apoptosis and anti-proliferation of non-small-cell lung cancer cells by modulating the differential expressions of superoxide dismutases and cell cycle G1 arrest. After C_8_-ceramide treatment, the SOD1 to SOD2 switch stimulated by ROS induces the excess accumulation of cyclin D1, a feedback loop, which in turn causes cell cycle G1 arrest. Eventually, C_8_-ceramide induces the growth arrest and the apoptotic cell death in lung cancer H1299 cells. The upwards arrows and downwards arrows indicate the upregulation and downregulation respectively.

In the study, C_8_-ceramide treatment may cause the switch of SOD1/SOD2 expression and the up-regulation of cyclin D1, which could sensitize NSCLC cells towards proliferation inhibition and apoptosis, and we herein presented a proposed model of the pathway accordingly (Figure 7). This model illustrated that the down-regulation of SOD1 was controlled by ROS, which resulted in negative feedback to SOD1, causing the excessive level of cyclin D1 in the H1299 cells. The up-regulation of ROS level and SOD2 were increased, and while the expression of SOD1 was decreased by the switching mechanism of SOD1/SOD2, which resulted in the up-regulation of cyclin D1 and the arrest of cell cycle G1 phase. Eventually, the cellular apoptosis is initiated, and the effector caspase-3 is activated, causing apoptosis of H1299 cells. The results of our study suggested that the anti-growth potential of exogenous long carbon-chain C_8_-ceramide against human non-small-cell lung cancer cells may occur through the modulation of the ratio of SOD1 and SOD2. Accordingly, the study suggested that C_8_-ceramide could be used for chemoprevention or chemotherapeutics of lung cancer treatment in future applications.

## 4. Materials and Methods 

### 4.1. Preparation of C_8_-Ceramide

C_8_-Ceramide (d-erythro-Sphingosine, *N*-Octanoyl) was purchased from Calbiochem-Behring Corp. (#219540, La Jolla, CA, USA). C_8_-ceramide was dissolved in DMSO (as stock solutions at 5 mM), and the aliquots were stored at −20°C before assays.

### 4.2. Cell Cultures

Human non-small-cell lung cancer H1299 cells (large cell carcinoma) were maintained in a DMEM medium (Invitrogen, Carlsbad, CA, USA) with 10% fetal bovine serum (FBS, Invitrogen, Carlsbad, CA, USA), 100 μg/mL streptomycin, 100 U/mL penicillin, 0.03% glutamine and 1 mM of sodium pyruvate. The cells were cultured kept at 37 °C in a humidified atmosphere with 5% CO_2_.

### 4.3. Cell Proliferation Assay

The proliferation rate was determined by a Trypan blue dye exclusion assay as previously described [57,58]. In brief, 1 × 10^5^ H1299 cells were seeded onto a 12-well culture plate. The cells were treated with C_8_-ceramide at indicated concentrations for 24 h respectively. Afterward, 0.2% Trypan blue was added to the wells. Finally, the viable cells were counted by Countess^®^ Automated Cell Counter (Invitrogen, San Diego, CA, USA). The assay was performed in triplicates, and the IC_50_ was calculated from the slope and intercept accordingly to the concentrations of C_8_-ceramide between the half-maximal proliferative inhibitions.

### 4.4. Apoptosis Assessment

Apoptosis was detected by Annexin/PI double staining as previously described with minor modifications [59]. Cells were treated with indicated concentrations of C_8_-ceramide for 24 h respectively. Cells were harvested and stained with 10 μg/mL of Annexin V-fluorescein isothiocyanate and 5 μg/mL of PI. Then cells were measured with a flow cytometer (FACSCalibur, Becton-Dickinson, Mansfield, MA, USA). Healthy cells (Annexin V^−^/PI^−^), the necrotic (Annexin V^−^/PI^+^), early apoptotic (Annexin V^+^/PI^−^) and late apoptotic cells (Annexin V^+^/PI^+^).

### 4.5. Cell Cycle Distribution

Propidium iodide (PI) (Sigma-Aldrich St. Louis, MO, USA) was used for assessing DNA content. In brief, cells were treated with indicated concentrations of C_8_-ceramide for 24 h respectively. After harvest, cells were fixed with 70% ethanol and washed with phosphate-buffered saline (PBS). After centrifugation, cells were incubated with 10 μg/mL RNase A and 10 μg/mL propidium iodide in PBS for 15 min at room temperature in the dark. The distributions of the cell cycle were analyzed using a BD LSRII flow cytometer (Becton Dickinson, San Jose, CA, USA).

### 4.6. Flow Cytometry-based ROS assessment

The changes in endogenous ROS levels were assessed using the redox-sensitive fluorescence indicator 2′,7′-dichlorofluorescein diacetate (DCFDA) (Sigma-Aldrich, St. Louis, MO, USA). The ROS assessment was described previously [56]. Briefly, cells were treated with or without C_8_-ceramide for 24 h respectively and then harvested and stained with the oxidative dye 100 nM DCFDA in PBS for 30 min at 37 °C. The measurement wavelengths for excitation and emission were 485 and 530 nm, respectively.

### 4.7. Wound Healing Assay

The cells were seeded and grown on a 12-well plate for overnight. Then cells were scratched by a 200 μL tip to generate a wound area. Cells were further incubated with medium containing 8% FBS media at 37 °C for 12 h for reconstructing the wound area. The wound areas were measured by a software TScratch (http://www.cse-lab.ethz.ch) [59].

### 4.8. Boyden’s Transwell Assay

The invasion ability was performed by a 12-well plate combined with inserts with polycarbonate filters (8-μm pore size). Briefly, the lower well contained 800 µL of medium containing 10% FBS. One hundred thousand H1299 cells in serum-free medium were seeded onto a transwell insert (Greiner) and were incubated for 16 h. Invaded cells were fixed with 4% paraformaldehyde and then stained with Giemsa (Merck). All stained cells were counted under a microscope (TE2000-U; Nikon, Tokyo, Japan)

### 4.9. Western Blotting Assay

In brief, cells were collected for lysate preparation. Forty μg of protein lysates were loaded and electrophoresed on 10% SDS-polyacrylamide gel and then transferred to nitrocellulose membranes, and then were blocked with 5% nonfat milk. Subsequently, the membranes were reacted with primary antibodies against SOD1 (GeneTex Co., Cat No. GTX100659, Irvine, CA, USA), SOD2 (GeneTex Co., Cat No. GTX116093, Irvine, CA, USA), cyclin D1 (GeneTex Co., Cat No. GTX112874, Irvine, CA, USA), caspase-3 (Imgenex, Cat No. IMG-144A, Imgenex, San Diego, CA, USA), β-actin (Santa Cruz Biotech, Cat No. #sc-8432, Santa Cruz, CA, USA). The membranes were further reacted with the corresponding secondary antibodies. The chemiluminescence detection kit (ECL^TM^, Amersham Piscataway, NJ, USA) was used for detecting specific proteins.

### 4.10. Statistical Analysis

All data of the study were presented as mean ± S.D. Differences between vehicle controls and experimental groups were analyzed by one-way ANOVA test.
